# Advances in the Application of Supramolecular Hydrogels for Stem Cell Delivery and Cartilage Tissue Engineering

**DOI:** 10.3389/fbioe.2020.00847

**Published:** 2020-07-21

**Authors:** Xin Yan, You-Rong Chen, Yi-Fan Song, Jing Ye, Meng Yang, Bing-Bing Xu, Ji-ying Zhang, Xing Wang, Jia-Kuo Yu

**Affiliations:** ^1^Knee Surgery Department of the Institute of Sports Medicine, Peking University Third Hospital, Beijing, China; ^2^Beijing National Laboratory for Molecular Sciences, State Key Laboratory of Polymer Physics and Chemistry, Institute of Chemistry, Chinese Academy of Sciences, Beijing, China; ^3^University of Chinese Academy of Sciences, Beijing, China

**Keywords:** supramolecular hydrogels, stem cell delivery, extracellular matrix, cartilage tissue, tissue engineering

## Abstract

Cartilage defects pose a great threat to the health of the aging population. Cartilage has limited self-regeneration ability because it lacks blood vessels, nerves and lymph. To achieve efficient cartilage regeneration, supramolecular hydrogels are used in medical applications and tissue engineering as they are tunable and reversible in nature. Moreover, they possess supramolecular interactions which allow the incorporation of cells. These hydrogels present great potential for tissue engineering-based therapies. This review presents advances in the development of stem cell-laden supramolecular hydrogels. We discuss new possibilities for stem cell therapy and their uses in cartilage tissue engineering. Gray areas and future perspectives are discussed.

## Introduction

Cartilage damage is commonly caused by trauma, sports injuries or arthritis. Normal cartilage lacks blood vessels, nerves, and lymph. In case of cartilage injury, self-healing of cartilage tissues is limited by inadequate nutrient supply, leading to the development of osteoarthritis (OA). In 2019, OA was reported to be the major cause of disability in people aged 45 and above, affecting over 303 million people worldwide (Kloppenburg and Berenbaum, 2020). The mainstay clinical treatments for cartilage injury include microfractures (bone marrow stimulation), cell transplantation, and osteochondral tissue transplantation. However, these methods are associated with side defects, rendering them unable to achieve satisfactory cartilage repair (Varela-Eirin et al., 2018). During bone marrow stimulation therapy fibrocartilage regeneration is induced rather than normal functional hyaline cartilage. The conventional cell transplantation treatments lacks a suitable carrier to protect the cells. Consequently, the cells die from the joint shear force or are diluted by the joint fluid, leading to weakened cell therapy efficiency. In addition, the osteochondral tissue transplantation suffer from limited donor supply, immune resistance, and insufficient integration (Yang et al., 2017). To overcome the above shortcomings, cartilage tissue engineering is applied to promote cartilage regeneration.

The extracellular matrix (ECM) is the main component of cartilage tissue and provides structural and biochemical support to chondrocytes. It is made up of mainly high content of water, protein fibers, and polysaccharides. Over the last few decades, hydrogels mimicking ECM have become the main scaffolds for reconstructing artificial three-dimensional biochemical and biophysical environments to regulate the fate and function of cells, such as directed differentiation of stem cells (Eslahi et al., 2016; Gan et al., 2019; Wang et al., 2020). Since biocompatibility is a prerequisite, only a few synthetic polymers such as polyethylene glycol, poly (lactic-co-glycolic acid), and natural polymers such as protein, DNA, polysaccharide, etc. can be used as the backbone of the hydrogel network (Seliktar, 2012; Kharkar et al., 2013). Although conventional hydrogels have many good properties, given the dynamic nature of tissue regeneration and integration with the host, their permanent covalent crosslinking is insufficient for tissue engineering applications (Rosales and Anseth, 2016). Recently, supramolecular hydrogels have received much attention as novel reversible cross-linking scaffold materials. They have inherent progressive features such as natural dynamic tissue simulation, injectability, self-healing, and adjustable mechanical behavior, providing great advantages in three-dimensional cell culture and tissue regeneration application (Du et al., 2015; Karoyo and Wilson, 2017; Shigemitsu and Hamachi, 2017; Hu et al., 2018). These properties are based on the dynamic nature of their composition, as well as the supramolecular bonding motifs from non-covalent supramolecular interactions such as hydrogen bonding, hydrophobic interactions, host-guest complexation, electrostatic interaction and metal-ligand coordination to crosslink (McCune et al., 2020). In cartilage regeneration therapy, the supramolecular hydrogel provides mechanical protection to living cells and cushions against damage from shear force in harsh knee environments, anchors cells to the targeted defect sites more efficiently, and protects them from elution by joint fluid (Xu et al., 2019). In addition, it provides a bionic three-dimensional growth environment for cells, conserves the phenotype of the chondrocytes, and develops normal hyaline cartilage from the deposition of extracellular matrix. Having self-healing and injectable properties, the supramolecular hydrogels can be delivered to target defect sites by intra-articular injection. Thus, they perfectly fill the irregular defective sites and integrate with the surrounding native cartilage tissue rather than freely shifting, establishing a minimal invasive and faster therapy (Hou et al., 2015; Marquardt and Heilshorn, 2016).

The development of stem cell therapy and different gelators for the synthesis of hydrogels has promoted intelligent design and synthesis of supramolecular hydrogels for cartilage tissue engineering. These hydrogels enhance 3D cell culture functions through self-assembling components or incorporating other reagents (Kim et al., 2011; Li G. et al., 2015). Once they integrate the additional properties, they achieve a versatile configuration and can address more specific requirements in cartilage regeneration therapy. In recent years, various hydrogel materials for cartilage tissue engineering based on stem cell therapy, such as agarose-based hydrogels (Salati et al., 2020), natural hydrogels (Bao et al., 2020), photopolymerizable hydrogels (Meng et al., 2019), and sulfated polysaccharide-based hydrogels (Dinoro et al., 2019). However, there are few reviews on advances in supramolecular hydrogels for cartilage tissue engineering and stem cell therapy. Given this, this review focuses on recent significant works on the development of supramolecular hydrogels for stem cell therapy and cartilage tissue engineering applications.

## Overview of Supramolecular Hydrogels

Supramolecular hydrogels, also known as “physical hydrogels,” differ from traditional “chemical hydrogels” mainly in their reversible and non-covalent intermolecular crosslinks. The polymer chains in traditional chemical hydrogels are cross-linked via covalent bonds. This endows them with high mechanical strength, structural stability, and shape memory. However, the robust covalent cross-linking increases brittleness, deprives them of self-healing properties, impairs their injectability. More importantly, this limits cell proliferation and migration, hence limited application in three-dimensional cell culture. Compared to traditional hydrogels, “physical hydrogels” rely on non-covalent supramolecular interactions based on self-assembly such as hydrogen bonding, ionic and associative interactions, host-guest complexation, metal-ligand complexation, and electrostatic interactions (Burnworth et al., 2011; Appel et al., 2012; Li et al., 2014). The dynamic nature of these interactions endows them with self-healing and shear thinning properties, hence effective cell delivery and survival. Therefore, supramolecular hydrogels have excellent injectability, simpler synthesis, multi-functional and flexible physical properties. According to the gelation mechanism, supramolecular hydrogel materials can be classified into those formed through directed molecular stacking or molecular recognition motifs (Webber et al., 2016).

Supramolecular hydrogels materials composed of two or more components are of particular interest. Hydrogelators are made up of multiple non-gelators components that combine to form a gel through intermolecular interactions. The multi-component hydrogels have intrinsic advantages over one-component hydrogels as the tunability of the components accords them versatility and dynamic reversibility. This allows high morphological diversity, better mechanical and unique synergistic properties. In addition, modifying the component structure or functionalizing one of the components allows the gelation process and the properties of the gel to be easily adjusted, which is good for diverse applications (Du et al., 2015). Accordingly, understanding the complex interactions between the organized supramolecular components is essential in understanding natural supramolecular self-assembly and dissociation mechanisms, promoting in-depth research in biomedical applications (Hu et al., 2018).

An ideal supramolecular hydrogel for cell delivery and cartilage tissue engineering should provide cells with a decent three-dimensional bionic growth microenvironment under physiological conditions and maintain stability after transplantation. Therefore, this review excludes the supramolecular hydrogels synthesized under the stimulation of catalysts or non-physiological pH levels unsuitable for cartilage tissue engineering. Physically, supramolecular hydrogel is a 3D dynamic cross-linked network with a lot of water, which acts as a soft, compliant hydration material. The dynamic cross-linking is primarily reflected in the transitional as well as dynamically alternating breaking and reforming state between the inner chains. The potential to form such dynamic bonds depends on the association dissociation reaction rate constant, the equilibrium constant, and the monomer concentration. Generally, the degree of association is affected by the equilibrium constant and the kinetic properties are affected by the rate constant (Rodell et al., 2015). In cartilage tissue engineering, shear-thinning and self-healing ability are the most outstanding properties of supramolecular hydrogels essential for cell delivery and survival. They are conferred by the physical dynamic cross-linking properties of the hydrogel components (Xu et al., 2019). Under shear stress, smart supramolecular hydrogel materials exhibit liquid-like properties, which facilitates the delivery of cells to target site via minimal invasive injection. Subsequently, the hydrogel self-heals reinstating its mechanical properties, ultimately completing the stable site delivery process of the encapsulated cells without leakage (Shao et al., 2017).

## Supramolecular Hydrogels for Stem Cell Delivery

An ideal tissue engineering hydrogel should promote cell infiltration, encapsulation, and deliver cells to target cells. It should also autonomously, quickly and repeatedly self-heal *in situ* under physiological conditions. But the premise of the above properties is superior compatibility with cells. In this section, recent advances in supramolecular hydrogels applied in three-dimensional stem cell culture which have not been applied in cartilage tissue engineering are reviewed according to their supramolecular interactions.

### Hydrophobic Interactions

Hydrophobicity imparts unusual properties to non-polar aqueous solutions and plays an important role in various chemical and biophysical phenomena, such as protein folding or self-assembly of amphiphilic molecules into micelles and membranes (Whitesides and Grzybowski, 2002). Hydrophobic interactions are unique non-covalent interactions that do not involve direct intermolecular attraction in their interactions. They are driven by the tendency of water molecules to keep their H-bond network intact around non-polar solutes (Otto and Engberts, 2003). The formed molecular rearrangements can result in complex colloidal behavior of amphiphilic molecules in aqueous solution. A polymer-based hydrogel with hydrophobic interactions is prepared by introducing a hydrophobic sequences at the end or inside the hydrophilic polymer chain. The resulting transient networks from the interchain interactions depend on the polymer concentration, polymer structure and the ratio of the hydrophobic components (Mihajlovic et al., 2018).

#### Peptide Amphiphiles

The innate biocompatibility properties of peptide makes it an excellent choice as a building block for supramolecular hydrogel. Peptide amphiphiles (PA) assembled through covalent binding of one or more peptides to an intelligent designed hydrophobic synthetic polymer have been widely used to prepare self-assembled, bioactive supramolecular hydrogels (Fichman and Gazit, 2014). PA hydrogels have attracted extensive research attention in 3D cell culture applications due to their biodegradable and non-toxic properties. For instance, Inostroza-Brito et al. (2015) reported a dynamic system formed by conformational modification of elastin-like proteins (ELP) by peptide amphiphiles. This system formed a robust membrane that exhibited controllable assembly and disassembly abilities, adhesion and sealing to surfaces, self-healing, and generation of tubular structures in a highly spatiotemporal controlled configuration. Mouse adipose primary stem cells (mADSC) were successfully cultured in the ELP/PA tube material for 21 days. During the cultivation process, the cells demonstrated high adhesion and diffusivity on the outer surface of the test tube and grew in multiple layers. In addition, their metabolic activity was consistently good, and their viability was the same as cells seeded onto tissue culture plastic. To explore the amyloid-based hydrogels applicability as scaffolds for stem cell differentiation in neuronal cell line, Jacob et al. (2015) designed a series of peptides based on the Aβ42 high aggregation tendency C-terminus of the Alzheimer’s disease. These Fmoc-protected peptides self-assembled into β-sheet-rich nanofibrils and formed thermally reversible, non-toxic and thixotropic hydrogels. Compared to existing peptide hydrogels, the amyloid-based hydrogels are favorable not only due to their thixotropy, but since the gel properties can be customized by altering the amino acid sequences and/or environmental conditions. According to detailed studies, this gel initiates neuronal differentiation of human mesenchymal stem cells (hMSCs) by providing fibril-mediated contact guidance. Moreover, self-assembled PA supramolecular hydrogels have been explored for co-delivery of dental stem cells and growth factors to regenerate dentin (Galler et al., 2012), and for angiogenesis (Hosseinkhani et al., 2006).

#### Amphiphilic Block Copolymers

Block copolymer is a copolymer with a specific chemical structure and molecular weight that can be synthesized as per the requirements. It is characterized by the presence of two or more different structure segments in a single linear molecule. The block copolymers with amphiphilic nature self assembles into specific supramolecular ordered aggregates, such as micelles, vesicles or fibers, in a solution. When dissolved in water, the amphiphilic block copolymer spontaneously forms a polymer micelle with a hydrophilic shell and a lipophilic core. Zhang et al. (2011) reported a degradable self-assembled hydrogel based on the physical association of amphiphilic branched poly (ethylene glycol) -poly (propylene sulfide) (PEG-PPS) block copolymers. The PEG-PPS hydrogels successfully applied for the delivery and promotion of human-induced pluripotent stem neural progenitor cells (iPS-NPC)as an injectable biomaterial. In addition, PEG-PPS allows angiogenesis and at the same time selectively inhibits astrocyte infiltration. This hydrogel system can be further used in numerous tissue engineering regeneration applications. By integrating the required signals such as proteins, growth factors and RNA, supplementary potential abilities can be achieved.

### Host–Guest Interactions

Host–guest interactions are specific non-covalent interactions that have been widely applied in the construction of supramolecular hydrogels. These interactions are based on selective inclusion complexation between macrocyclic hosts, such as cucurbiturils (CB), cyclodextrin (CD), crown ethers, calix[n]arenes, and smaller guest molecules. The host selectivity for the guest is not just a simple hole-fitting concept, it can be affected by solvent, multiple binding sites or secondary interactions (Schneider and Yatsimirsky, 2008). Self-healing supramolecular hydrogels from inclusion complexation can be prepared by mixing polymers with host and guest or copolymerizing prefabricated host–guest inclusion complexation with co-monomers (Yang et al., 2015). Although all macrocycles have the potential to construct self-healing hydrogels, CB and CD have been mainly used to build supramolecular hydrogels for cell delivery. Thus, CB and CD are reviewed in this study.

#### Cyclodextrin (CD)

**FIGURE 1:**
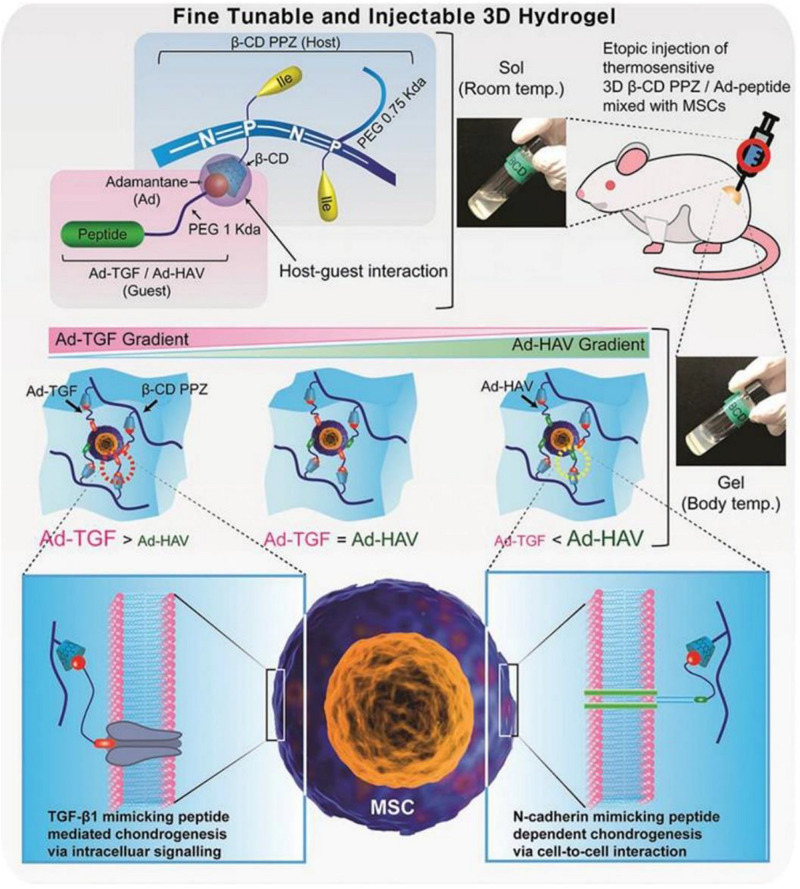
Schematic diagram of mesenchymal stem cells (MSCs) encapsulated with 3D β-cyclodextrin poly(organophosphazene) (β-CD PPZ), adamantane-TGF, and HAV (Ad-TGF and Ad-HAV). The above molecules and cells were mixed to form a 3D thermosensitive hydrogel. After their injection, it was possible to convert the liquid into a gel state at body temperature. The fate of the MSCs can be controlled by varying the stoichiometric ratio of the different molecules. Adapted with permission from Hong et al. (2019).

Cyclodextrin is a family of cyclic oligosaccharides that are made up of glucopyranose subunits linked by α-(1,4). The subunits are composed of α-CD, β-CD, and γ-CD which contain six, seven, and eight glucose molecules, respectively. CD acts as a host molecule by forming an inclusion complex with a specific guest via its hydrophobic cavity (Huang et al., 2020). In a recent study, Hong and Song (2019) designed a thermosensitive poly(organophosphazene) loading β-CD (β-CD PPZ, host) and adamantane elongated with Arg-Gly-Asp (Ad-RGD, guest). The guest molecules AD and RGD were linked to biocompatible PEG to avoid steric hindrance between MSCs and β-CD PPZ. Based on the application of Ad-RGD controlled concentration *in vitro* and *in vivo*, regulated MSCs behaviors were induced. As the content of Ad-RGD in β-CD PPZ hydrogel increased, the survival rate of MSCs increased and the expression of osteogenic factors were elevated. In contrast, reducing the Ad-RGD resulted in reduced viability and adipogenic capacity of the MSCs. This confirmed that a host-guest interaction system with a thermosensitive 3D hydrogel can control the survival and differentiation of MSCs by varying guest molecules. Similarly, injectable 3D hydrogels constructed with β-CD PPZ and two guest molecules (Ad-TGF and Ad-HAV) were reported (Figure 1). Their gelling properties were maintained at body temperature. Injection of MSCs encapsulated in this fine-tuned hydrogel triggered various chondrogenic differentiation stages in 3 weeks post-injection by the stoichiometrically strict control of Ad-peptides based on host–guest interaction. Thus, simply changing the adamantane-bearing peptide and its stoichiometry, the adjustable and injectable 3D hydrogel can be used as a platform technology for on-demand stem cell niche (Hong et al., 2019).

#### Cucurbiturils (CB)

**FIGURE 2:**
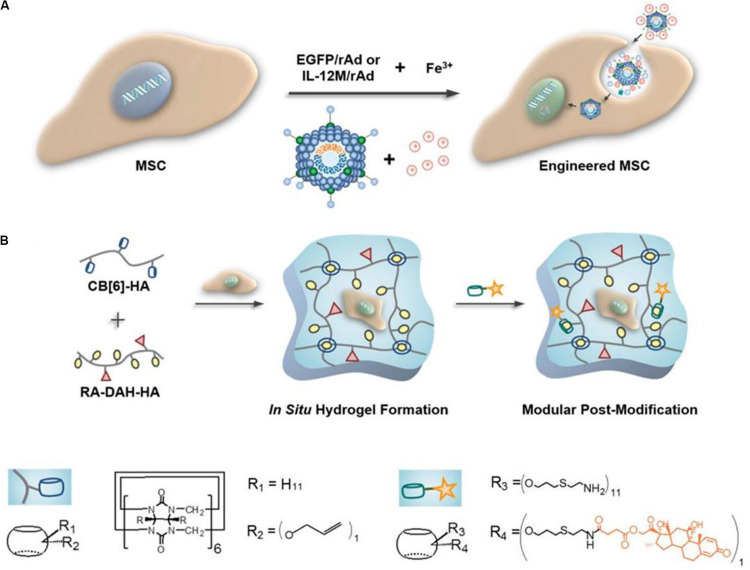
Schematic diagram of engineered mesenchymal stem cells (eMSCs) therapy using supramolecular hyaluronic acid (HA) hydrogel. **(A)** Under ferric ions, Enhanced Green Fluorescent Protein (EGFP) or interleukin-12 mutant transgenes were transfected into MSCs via recombinant adenoviral (rAd) vectors. **(B)** Dexa-CB[6]/RA-DAH-HA hydrogel was synthesized by mixing CB[6]-HA solution and RA-DAH-HA solution with engineered MSCs and modular modification. Adapted with permission from Yeom et al. (2015).

The size of CB cavity size is the same as that of CD. However, unlike CD, it has two identical cavity entrances with carbonyl edges. Macrocyclic compounds made up of glycoluril units, cucurbiturils (CB[n], *n* = 5–8, 10; *n* = 14 the most abundant) can form binary 1:1 or ternary 1:1:1 host-guest complexes with multiple guest molecules. The cavity of CB is sufficiently large to accommodate two guests (Cheng et al., 2013). Nevertheless, chemical functionalizing CB is challenging, and the controllable difficulty results in several multi-arm functionalized CB mixture. Due to the complex characteristics above, the suitably functionalizing CB possess multi-biological applications functions. Recently, Fink et al. (2020) explored the cytotoxic effects of different CBs on HaCaT keratinocytes and erythrocytes. At high concentrations (30 mg/mL), CB[5] and CB[6] were non-cytotoxic, while incubation with CB[7] at a low concentration (3.75 mg/ml) induced apoptosis. All the CBs studied did not have hemolytic effects on erythrocytes. These results suggest that CB has a great potential as a host complex in cell delivery applications. Moreover, taking advantage of the selectivity and strong host-guest interactions between CB[6] and polyamines, Yeom et al. (2015) developed a supramolecular HA hydrogel with controllable crosslink density and good physical properties. They used cucurbit[6]uril-conjugated hyaluronic acid (CB[6]-HA) and diaminohexane conjugated HA (DAH-HA), which were able to stay stable in the body for more than 11 days (Figure 2). This supramolecular HA hydrogel has a good biocompatibility, and its physical and chemical properties can be regulated by switching the guest molecules. Besides, engineered mesenchymal stem cells (eMSCs) encapsulated in hydrogel can survive in mice for more than 60 days. The above outcomes confirm that of supramolecular HA hydrogel have a great application potential as 3D artificial ECM in cell delivery and cartilage engineering.

### Hydrogen Bonding

Another strategy of fabricating a self-healing reversible network is by introducing complementary hydrogen bond donor and acceptor motifs into dynamic supramolecular polymer building blocks. Hydrogen bonding is regarded as a crucial self-assembly process since it participates in DNA and RNA nucleobase pairing as well as protein assembly naturally (Shao et al., 2017). A single hydrogen bond strength is weaker than the covalent bonds and some other non-covalent bonds. However multivalent bonding significantly increases the degree of association within the supramolecular and increase the bonding strength. In addition, hydrogen bonds participate in the synthesis of some tough covalent materials such as silk, through synergy (Keten et al., 2010).

#### Amino Acids and Peptide

**FIGURE 3:**
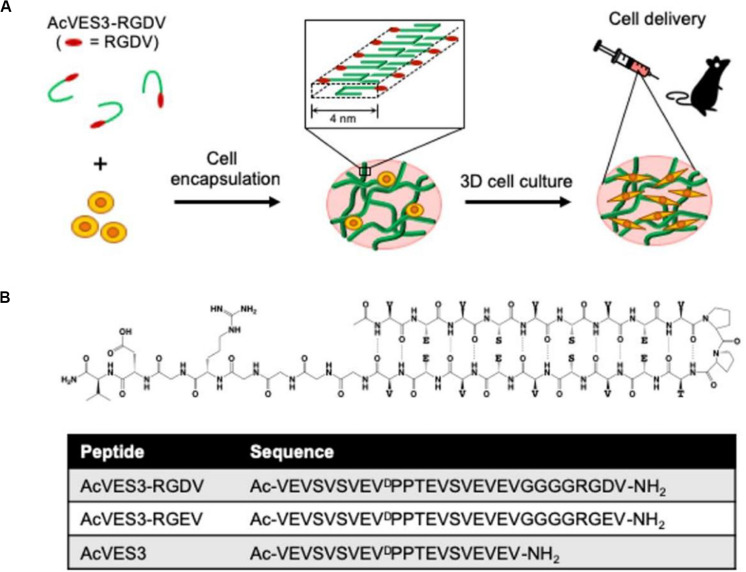
Design of a negatively charged fibril hydrogel composed of self-assembled peptide AcVES3-RGDV. **(A)** Cell encapsulation during peptide self-assembly formed a fibril-rich hydrogel loaded with syringe injectable cells to target tissues. **(B)** Schematic diagram of the asymmetric AcVES3-RGDV hairpin peptide and the sequence of the used peptides. Adapted with permission from Yamada et al. (2019).

Amino acids and peptide derivatives are a class of supramolecular gelling agents that have been systematically studied. The main driving force for their gelation is the intermolecular hydrogen bonds between amide bonds. These directional and strong amide hydrogen bonds promote the effective self-assembly of peptides in aqueous solutions. In addition to the amide bonds, other functional groups, such as carboxylic acid, hydroxyl, pyridine, urea and ureidopyrimidinone (UPy), provide multiple hydrogen bonds to enhance the gelling ability once introduced into the gelling agent structure. The peptide hydrogel can adjust the porosity and stiffness of the gel network by altering its local nanostructure. Besides, the peptide hydrogel structure remains stable when exposed to either solutions or body fluids. This is an important prerequisite for stem cell delivery and cartilage tissue engineering. Recently, Yamada et al. (2019) designed a negatively charged fibril hydrogel composed of self-assembled peptide AcVES3-RGDV (Figure 3). In the process of the peptide self-assembly, cells were successfully encapsulated. The gelation process was induced by adjusting the ionic strength or temperature of the solution, avoiding large changes in pH. The cells stably adhered to the AcVES3-RGDV gel network, achieving long-term *in vitro*/*in vivo* 3D cell culture. The gel exhibited good shear-thinning and self-healing properties and was injectable in a minimally invasive way. Similarly, a variety of self-assembled peptide hydrogels with excellent shear-thinning and self-healing properties have been reported as potential candidates for stem cell delivery (Smith et al., 2016; Tang et al., 2019).

#### Deoxyribonucleic Acid (DNA)

DNA is considered to be an excellent building block for multi-functional smart materials. According to the Watson-Crick base pairing principle, DNA polymer chains can self-assemble into secondary or higher-level structures through hydrogen bonding in a physiological environment. Moreover, DNA molecules are highly considered as promising materials due to specific base-pair recognition, designable sequences, and predictable secondary and advanced structures. In 2009, Liu D and his research team successfully synthesized a novel pure supramolecular hydrogel based on DNA self-assembly using either short duplexes or i-motif structures. The biocompatibility and permeability of the DNA hydrogels were verified by placing a single cell in microwells and thereafter sealing with DNA hydrogels. The results revealed that small molecular nutrients reached the cells very quickly via the gel network, promoting cell growth (Jin et al., 2013).

**FIGURE 4:**
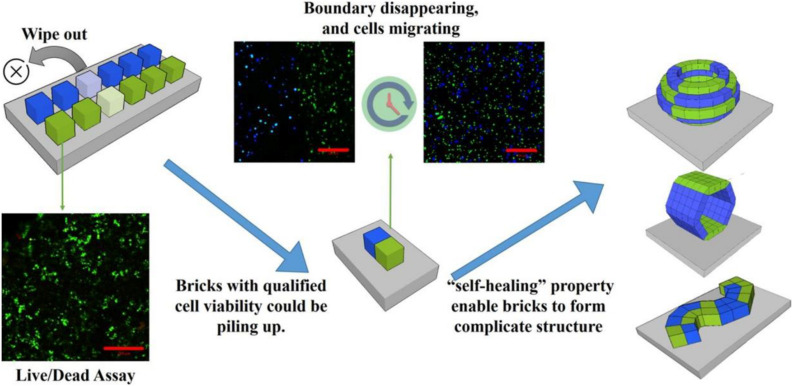
Schematic illustration for the construction of 3D tissue-like structures using brick-to-wall technology. Different types of cells were 3D cultured in each DNA hydrogel brick for some time to eliminate poorly viable cells. Thereafter, the excellent self-healing DNA hydrogel was used to splice each brick into the pre-designed structure. Adapted with permission from Wang et al. (2017).

Subsequently, they developed a new “brick-to-wall” technology based on the exceptional characteristics of the DNA supramolecular hydrogels to create 3D tissue-like structures (Figure 4). They encapsulated different types of cells including suspended cells and adherent cells into DNA hydrogel bricks, and then built the bricks into 3D structures. The results demonstrated that the cells could effectively migrate between the DNA hydrogel bricks and grow (Wang et al., 2017). This new hydrogel combines dynamic and strong mechanical strength and good biocompatibility with excellent molecular permeability (Cheng et al., 2009). Besides, DNA hydrogel has been successfully used in 3D cell printing and thus is considered the most promising material for 3D cell culture and tissue engineering (Li C. et al., 2015).

**FIGURE 5:**
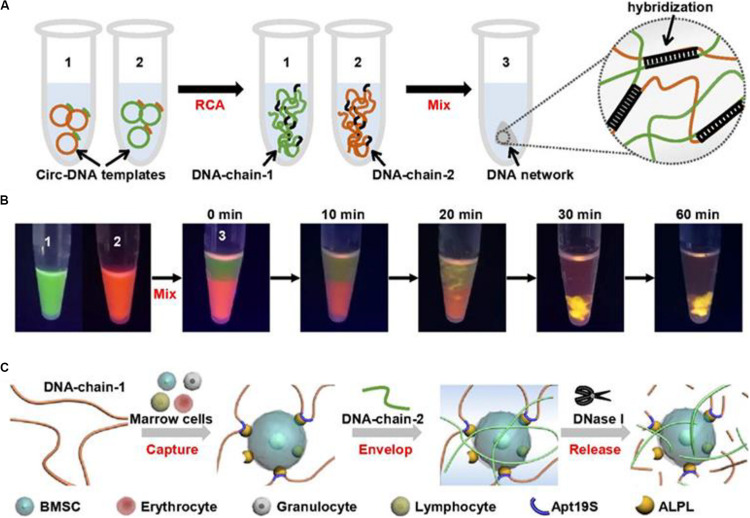
Schematic diagram of physically cross-linked DNA hydrogel for capturing stem cells. **(A)** The 3D DNA hydrogel network synthetic process through rolling circle amplification. **(B)** To visualize the formation process of DNA hydrogel network, DNA chain 1 and DNA chain 2 were stained with SYBR green and gel red, respectively. **(C)** The process BMSCs capturing, encapsulating, and releasing by the DNA hydrogel. Adapted with permission from Yao et al. (2020).

In a recent study, Yang D and his group developed a DNA network-based cell fishing strategy. This was effective in bone marrow mesenchymal stem cells (BMSCs) capture and provided a good microenvironment for 3D cell culture (Figure 5). The DNA network is constructed via self-assembly of two ultra-long DNA strands through a double rolling circle amplification method. The aptamer sequence in DNA-chain-1 specifically attaches to the bone marrow BMSCs. Notably, BMSCs can be released without damaging the DNA network through enzymatic hydrolysis. This design provides an effective strategy for fishing target stem cells from a mixture with non-target cells (Yao et al., 2020).

#### 2 Ureido-4[1H]Pyrimidinone (UPy)

During the 1990s, Meijer and his group described quadruple hydrogen bonding in building blocks 2-ureido-4[1H]pyrimidinone (UPy). This has been widely adopted as the force behind the development of supramolecular hydrogels (Mol et al., 2019). Multiple hydrogen bonding units simultaneously act as hydrogen bond donors and acceptors. This increases the concentration of hydrogen bonding motifs in the solution thus enhancing the degree of association between supramolecular building blocks. Recently, the preparation of a biocompatible UPy-based hydrogel has been accomplished. The hydrogel can spontaneously self-assemble under physiological conditions and is applied for BMSCs delivery and tissue engineering (Hou et al., 2015). Liu et al. (2019) utilized a bioactive gelatin methacrylate (GelMA) with 2-(2-methoxyethoxy)ethyl methacrylate (MEO2MA) and UPyMA to develop hybrid branched copolymer. When the temperature rises above the critical solution temperature (LCST) of the supramolecular copolymer there is rapid gelation. During the gelation process, the PMEO2MA fragments are dehydrated and assemble into clusters, forming an hydrophobic microenvironment. This promotes UPy polymerization into chains and finally, a crosslink network of four hydrogen bonds is formed. The BMSCs encapsulated in this hydrogel maintain high viability. *In vivo* studies have revealed that the hydrogel formed *in situ* provides physical protection to the BMSCs against mechanical damage, allowing the intact cells to subcutaneously survive for 3 weeks. This approach offers a great biological platform for stem cell delivery with excellent cell retention capacity.

### Electrostatic Interactions

Ionic interactions are used to enhance the performance of materials since relatively low concentrations of ionic groups are required to adjust the mechanical, physical, and dynamic properties of polymers. Among them, copolymers of less than 15 mol% ionic groups and polyelectrolytes have been widely used to construct ionic supramolecular crosslinking networks (Strandman and Zhu, 2016). Recently, Zhong W and his group designed a supramolecular hydrogel by assembling cell adhesive peptide conjugate (Pept-1) and alginate (ALG). Compared to the Pept-1 gel alone, the electrostatic interaction and metal chelation-mediated assembly method afforded the composite hydrogel with better mechanical strength and denser nanofibril structure. The Pept-1/ALG hydrogel presented with excellent injectability, thixotropy, and biocompatibility, promoting adhesion and migration of fibroblast cells *in vitro* (Zhai et al., 2019). ECM not only provides abundant biologically active molecules for cell proliferation, migration and differentiation, but also affects cell behavior and function through the intertwined collagen fibers. Inspired by the functional structure of ECM, Chen Xiaoyuan’s group synthesized biomimetic molecules that could self-assemble into layered nanofibrils hydrogel in a physiological buffer under the synergy of electrostatic interactions and van der Waals force. They successfully self-assembled new biomaterials mimicking the ECM structure and affecting the behavior of neural stem cells (NSCs) hierarchically by using pure bioactive molecules (Wang et al., 2016).

## Supramolecular Hydrogels for Cartilage Tissue Engineering

A comprehensive review of recent advances in cartilage tissue engineering based on scaffolds combined with different genetically modified MSCs is found in our previous work (Yan et al., 2019). In this section, the focal point is the latest research progress in supramolecular hydrogels in cartilage tissue engineering applications. However, most of the work has remained at the *in vitro* cell culturing level, hence far from regenerating damaged tissues or organs. Notably, research on the complex biological processes during cartilage repair in a dynamic environment is at initial stages. This has led to difficulty in designing tissue engineering hydrogel. In addition, most current supramolecular hydrogels are made up of only one or two molecular units. Thus, no hydrogel system can completely mimic the ECM of cartilage. This limits their role only as a supplement for internal cartilage repair. To solve these challenges, it is necessary to review the recent research progress of supramolecular hydrogels in the field of cartilage tissue engineering.

### Dextran–UPy Hydrogel

As opposed to covalently cross-linked hydrogels, supramolecular hydrogels are self-assembled through highly specific physical interactions, manifested as dynamic transient cross-linked hydrophilic polymer chains. Intelligent transient supramolecular cross-linking dissociates under mechanical stimulation, and reconstructs after stimulation is withdrawn. This unique property provides unparalleled advantages over other hydrogels. A prepared hydrogel can be injected in a minimally invasive way to seamlessly fill the lesions of different shapes, such as extensive irregular cartilage defects in OA, and to self-recover the gel state. This prevents sedimentation and leakage of stem cells and their functional biomolecules. If a supramolecular hydrogel with fast self-integration characteristics has an excellent biocompatibility and biodegradability, it is a promising solution for the regeneration of multiphase tissue complexes. It easily integrates hydrogel bricks carrying specific cells and signaling biomolecules into a multi-phase tissue structure. In this multi-phase hydrogel system, each phase regenerates in a region defined by its own space and integrates seamlessly at its interface.

**FIGURE 6:**
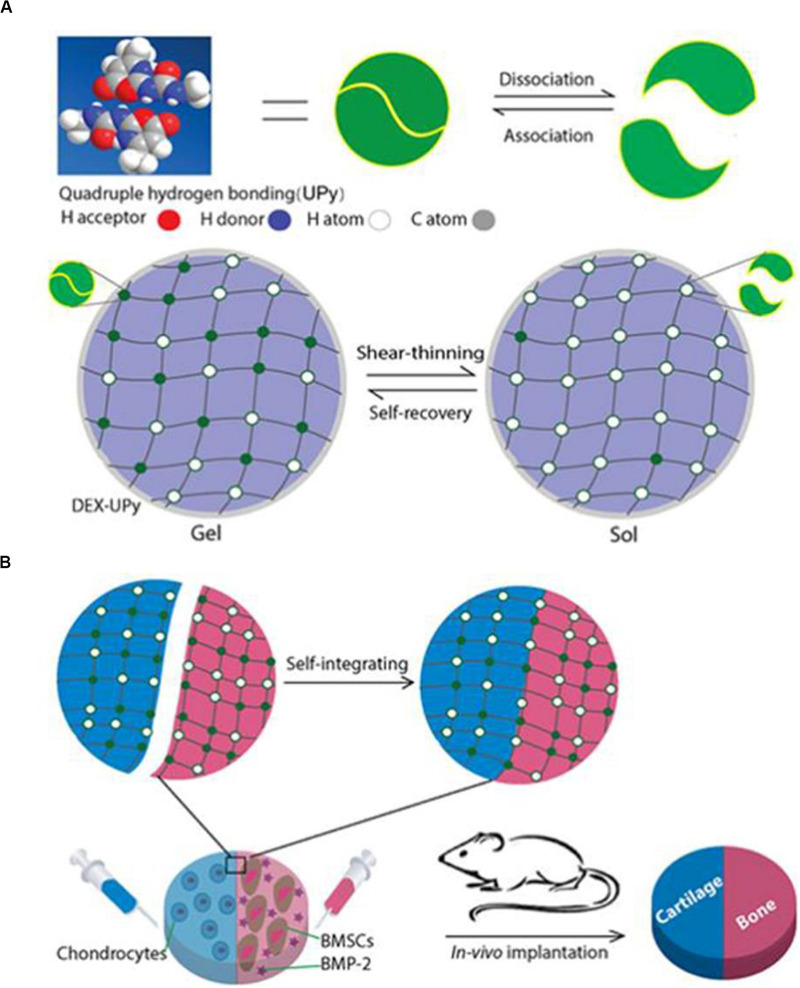
DEX-UPy hydrogel for composite tissue regeneration. **(A)** Schematic diagram of DEX-UPy hydrogel synthesis, shear-thinning, and self-healing properties. **(B)** Schematic diagram of self-integration of composite hydrogel and regeneration of cartilage-bone tissue complex after subcutaneous transplantation in nude mice. Adapted with permission from Hou et al. (2015).

To achieve the above, Ma PX and his group grafted many multi-hydrogen bond units onto biocompatible hydrophilic polymers. They were able to develop a novel rapid self-integration and shear-thinning supramolecular hydrogel, and to verify the regeneration of cartilage-bone tissue complex in a subcutaneous implantation model of nude mice (Figure 6). They chose UPy, the quadruple hydrogen bond unit, as it has higher intermolecular bond strength than single hydrogen bonds. The polysaccharide polymer dextran (DEX) approved by the FDA served as the backbone network. Through the hydroxyl groups on DEX, multiple UPy units were grafted onto DEX to form a polymer containing UPy. By changing the ratio of UPy to dextran, the graft density of UPy could be easily controlled. At optimum UPy density (DS) (DS 5.5, 10% w/w), the DEX-UPy polymer forms a stable hydrogel. After being loaded into the syringe, the hydrogel is liquid-like under the shear stress during the injection process, and solidifies rapidly after injection. The live-dead assay verified that both BMSCs and chondrocytes maintained high activity after being cultured in the hydrogel for 2 weeks. *In vivo* assay of subcutaneous transplantation in nude mice, chondrocytes for chondrogenesis and BMSCs plus bone morphogenetic protein 2 (BMP-2) for osteogenesis were encapsulated in two parts of hydrogel respectively. The results show that the novel hydrogel can support the growth of bone and cartilage, and the regenerated cartilage and bone can be seamlessly integrated (Hou et al., 2015).

### Host–Guest Macromer Hydrogel

Human BMSC (hBMSC) have been widely used in cartilage regeneration due to their versatility and availability. However, given the high mechanical load at the cartilage defect sites and catabolic factors in the joint cavity, a many hBMSC cells die, which limits their capacity to repair damaged cartilage. The lack of functional carrier materials for hBMSC to cartilage defects to provide physical protection and the presence of chondrogenic factors such as kartogenin (KGN) and TGF-β1 leads to low survival rate of hBMSC and unsatisfactory differentiation. To overcome these challenges, Bian L and his group developed a unique gelatin supramolecular hydrogel through a novel host-guest macromonomer (HGM) method. The resulting hydrogel formed by host-guest interaction between oligomeric Ac-β-CDs and aromatic residues of gelatin exhibited enhanced physical and biological functions, including self-healing, mechanical elasticity, injectability in the gel state, shape adaptation, controlled release of hydrophobic small molecule and supporting cell retention (Feng et al., 2016). The prefabricated HGM hydrogel could be injected through the G18 needle to completely fill the cartilage defect volume and firmly adhere to the surrounding cartilage. When hBMSCs were cultured in 3D with HGM hydrogels for 14 days, more than 95% of the cells survived, and the cell morphology substantially expanded from the initial round morphology into a fusiform shape, indicating that the cells encapsulated in the HGM hydrogel can actively interact with the surrounding hydrogel structure. Next, they examined the chondrogenesis of hBMSC in HGM hydrogel rich in KGN or TGF-β1. After 14 days of *in vitro* induction and 28 days of *in vivo* nude mouse subcutaneous transplantation culture, the expression of chondrogenic markers of hBMSCs were significantly increased. They further evaluated cartilage regeneration in a rat knee cartilage defect model. HGM hydrogel was injected and pressed fit into the cartilage defect. After 6 weeks, fully regenerated cartilage was observed in the defect site, which was white and smooth in appearance, well integrated with surrounding cartilage tissue, and close to healthy hyaline cartilage. Their studies demonstrated that compared to traditional chemically crosslinked gelatin hydrogels, HGM hydrogels strengthen the chondrogenesis of encapsulated hBMSCs. Moreover, the injected HGM hydrogel loaded with hBMSCS forms high-quality new cartilage in the rat knee cartilage defect model, indicating the great potential of HGM hydrogel carrier material for cells and drugs for cartilage tissue engineering (Xu et al., 2019).

### Supramolecular GAG-Like Glycopeptide Nanofiber Hydrogel

**FIGURE 7:**
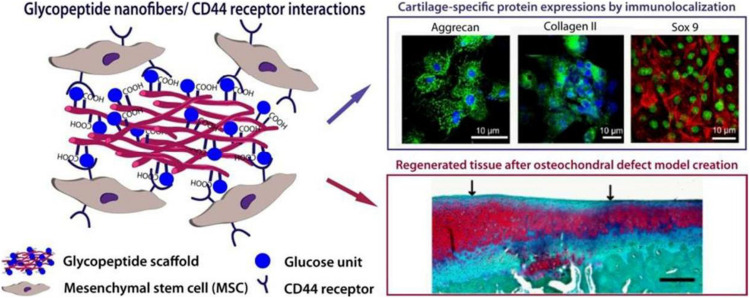
Design of a supramolecular GAG-like self-assembled glycopeptide nanofibers to induce chondrogenesis and cartilage regeneration. Glucose residues form the supramolecular GAG-like system through the self-assembly of peptide amphiphilic molecules (PA). Glycopeptide nanofibers promote the chondrogenic differentiation of MSCs by interacting with the CD44 receptor. Adapted with permission from Ustun Yaylaci et al. (2016).

Peptide amphiphilic molecules possess the structural properties of amphiphilic surfactants with biologically active peptides and unique nanostructure characteristics. They are highly biocompatible and biodegradable, hence have been extensively studied. A variety of non-covalent interactions jointly determine their self-assembly process, forming high aspect ratio nanofibers controllably. O Guler M and his group developed a glycopeptide nanofiber system designed to be used as a substitute of hyaluronic acid (HA) (Figure 7). This system is composed of Serine-linked β-D-glucose containing amphiphilic glycopeptide and PA with carboxylic acid. The self-assembly of glycopeptide amphiphilic molecules results in multiple glucose residues being closely arranged on the nanoscale supramolecular polymer system. *In vitro*, it was observed that self-assembled glycopeptide nanofibers recognize MSCs through the CD44 receptor and induce the chondrogenic differentiation in a manner comparable to natural HA. The glycopeptide nanofiber hydrogels were used to treat osteochondral defects model *in vivo.* They found that the hydrogels promoted the formation of hyaline cartilage instead of fibrocartilage. Based on the complete regeneration system provided by the hydrogel, the structure of the hydrogel matrix itself maintains a large number of MSCs at the defect site by promoting the adhesion of MSCs migrating from the bone marrow after microfractures. In this way, they enhance early mechanical stability at the defect site by stabilizing blood clots. These results show that the designed HA supramolecular analog can interact with mesenchymal stem cells through CD44 receptors, promoting cartilage lineage differentiation independent of exogenous growth factors. It offers the possibility to replace natural sources of HA and avoid the potential health hazards associated with natural HA scaffolds. Glycopeptide nanofibers can also be used as a less invasive and cell-free *in situ* cartilage regeneration materials (Ustun Yaylaci et al., 2016).

## Conclusion and Perspectives

In conclusion, the unique dynamics of smart supramolecular hydrogels and their shear- thinning and self-healing properties have propelled hydrogel research to a new level. During the past decade, research on supramolecular hydrogels has experienced rapid development, including the application in 3D culture of various cell types and tissue engineering for dental and cardiovascular tissues. Unlike conventional covalently crosslinked hydrogels, dynamic transient physical crosslinking endows supramolecular hydrogels with unique shear-thinning and self-healing properties. Thus, the gel can be injected to fill irregular defects seamlessly, such as the multiple irregular cartilage defects of OA. Once injected, the gel state can be self-restored, which prevents the leakage and sedimentation of encapsulated cells. The above characteristics indicate that supramolecular hydrogel can be used for minimally invasive injection in the treatment of cartilage defects. Although currently available supramolecular hydrogels have been fully characterized, most of them have not been evaluated *in vivo* or even *in vitro*. Unlike, cartilage tissue engineering, it is challenging to improve the mechanical properties of supramolecular hydrogels. We pospoes that a composite cartilage tissue material with enhanced mechanical properties can be formed by combining a supramolecular hydrogel that mimics the ECM microenvironment of cartilage with a harder mesh scaffold material. This will yield a material with slower erosion rate, repeated self-healing under physiological conditions and the sustained release of drugs or biological factors. Further research on supramolecular hydrogels should aim to improve biocompatibility and host integration, such as potential host immune responses, and the ability to regenerate and completely replace lost or eroded cartilage tissue. This will ultimately improve patient prognosis and quality of life.

## Author Contributions

XY and Y-RC drafted the manuscript. Y-FS, JY, MY, B-BX, and JZ collected and sorted the information. J-KY and XW designed the conception and revised the manuscript. All the authors contributed to the article and approved the submitted version.

## Conflict of Interest

The authors declare that the research was conducted in the absence of any commercial or financial relationships that could be construed as a potential conflict of interest.
